# A Combination of Aerobic Exercise at Fatmax and Low Resistance Training Increases Fat Oxidation and Maintains Muscle Mass, in Women Waiting for Bariatric Surgery

**DOI:** 10.1007/s11695-022-05897-1

**Published:** 2022-01-20

**Authors:** Inés Picó-Sirvent, Agustín Manresa-Rocamora, Adolfo Aracil-Marco, Manuel Moya-Ramón

**Affiliations:** 1 Department of Sport Sciences, Sport Research Centre, Miguel Hernández University, 03202 Elche, Spain; 2 Institute of Health and Biomedical Research (ISABIAL-FISABIO Foundation), Miguel Hernández University, 03010 Alicante, Spain; 3 Instituto de Neurociencias, UMH-CSIC, Miguel Hernández University, 03550 San Juan de Alicante, Spain

## Abstract

**Purpose:**

There is no consensus on the best exercise recommendation for women affected by severe obesity while they are waiting for bariatric surgery. For this reason, the effects of a combination of aerobic exercise performed at the intensity at which maximal fat oxidation is reached (Fatmax) with low-intensity resistance training were studied.

**Materials and Methods:**

Twenty sedentary middle-aged Caucasian women (43.2 ± 7.5 years, BMI = 46.5 ± 5.9 kg·m^−2^) were allocated to a control group (CG, *n* = 10) that followed solely the conventional preoperative care or to an experimental group (EG, *n* = 10) that, in addition, performed a 12-week individualized and supervised physical activity program (PAP) that combined aerobic training at Fatmax with low-intensity resistance training.

**Results:**

After the PAP, maximal fat oxidation during exercise increased in the EG (0.187 ± 0.068 vs 0.239 ± 0.080 g·min^-1^, *p* = 0.025, pre *vs.* post, respectively), but resting fat oxidation did not (0.088 ± 0.034 vs 0.092 ± 0.029 g·min^-1^, *p* = 0.685, pre *vs.* post, respectively). Additionally, the resting metabolic rate in the EG was also unchanged (1869 ± 406 *vs.* 1894 ± 336 kcal; *p* = 0.827, pre *vs.* post, respectively), probably because of the effects of resistance training on the maintenance of fat-free mass. No significant changes were observed in the CG.

**Conclusion:**

A PAP that combines aerobic exercise at Fatmax with low resistance training may counteract some of the deleterious side effects of the standard presurgical care of women waiting for bariatric surgery and increase maximal fat oxidation during exercise.

**Graphic Abstract:**

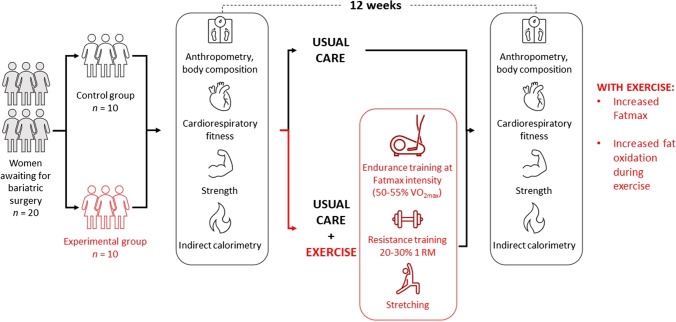

## Introduction

The existing obesity pandemic we live with today continues to increase and represents a serious public health challenge worldwide, especially due to the huge growth of class II and III obesity during these last years [1]. The concomitant coincidence of cardiometabolic risk factors with extreme obesity worsens the patient’s prognosis, and in these cases bariatric surgery (BS) is commonly recommended with the aim to quickly reduce the associated morbidity and mortality [2].

Although BS candidates are usually encouraged to increase their physical activity before the surgery, the effects of exercise programs delivered pre-operatively are still under discussion. Two recent meta-analyses [3, 4] coincide in that the very limited number of published works in which pre-operative exercise programs were prescribed to BS candidates makes almost impossible to clearly conclude their effects and their clinical relevance. Additionally, the heterogeneity between studies seems to be another major problem and it has been claimed that more evidence is needed to recommend specific training programs in terms of volume, intensity, frequency, and type of exercise in BS candidates [5]. Combining aerobic exercise with resistance training could be the best choice for the management of persons with obesity, given the summation of their effects on weight and fat loss with lean mass preservation, increased muscle strength, and cardiorespiratory fitness and prevention of additional comorbidities [6, 7]. In agreement with that, recent studies have shown that concurrent training, i.e., in which high intensity interval training (HIIT) and resistance training were combined during the same session, improves maximal oxygen uptake (VO_2_max), reduces fat mass (FM), increases fat-free mass (FFM), and reduces cardiometabolic risk factors in BS candidates [8, 9]. These positive effects may have also been extended to a better blood pressure control, a lower insulin resistance, and lower intrahepatic fat deposits [10].

Obesity is commonly considered a consequence of an energy imbalance that negatively affects energetic substrate metabolism, since the increase of body fat leads to changes in the physiologic function [11]. For example, energy expenditure is reduced in adults with severe obesity (BMI ≥ 35 kg·m^−2^) during rest and low-intensity exercise (i.e., performed at an intensity of 25 W) compared to patients with class I obesity or normal weight adults [12]. Similarly, obesity seems to be associated with metabolic inflexibility during exercise, which is characterized by reduced fat oxidation by the skeletal muscle in fasting conditions as well as by a reduced capacity to oxidize carbohydrates in response to food intake or in hyperinsulinemic state [13]. However, although obesity could imply lower fat oxidation rates, most of the previously published studies do not provide information in that regard during exercise. Moreover, there is a lack of knowledge regarding if physical activity or exercise could influence the metabolic rate and/or fat oxidation at rest in the specific context of BS.

The maximal quantity of fat that the organism can oxidize to obtain energy is called maximal fat oxidation (MFO) and the exercise intensity at which it occurs (typically medium intensities located around 45–55% VO_2_max in obese sedentary adults [14, 15]) is known as Fatmax. In overweight middle-aged women, training at Fatmax has shown improvements in body composition related to fat mass, blood lipid profile, cardiovascular function, and whole-body fitness after 10 weeks of intervention [16, 17]. In addition, a similar intervention in a recent study in overweight older women showed improvements in body composition and functional capacity compared to a control group [18]. Another study in which Fatmax training was combined with a low-fat diet showed weight loss in obese adults who followed a 1-year intervention compared to non-interventional controls [19]. However, to the best of our knowledge, isolated Fatmax training in patients with class II and class III obesity has only been performed in men, showing improvements in cardiorespiratory fitness and fat oxidation rates during exercise [20].

Given the fact that most of the BS candidates are women [21], and that their fat oxidation response to exercise is unknown, in the present work, we have measured the effect of a 12-week physical activity program that combined aerobic training at Fatmax with low-intensity resistance training on body composition, cardiorespiratory fitness, and substrate oxidation (both at rest and during exercise), in middle-aged women awaiting BS. We hypothesized that training at Fatmax would increase maximal fat oxidation during exercise.

## Materials and Methods

### Participants

**Fig. 1:**
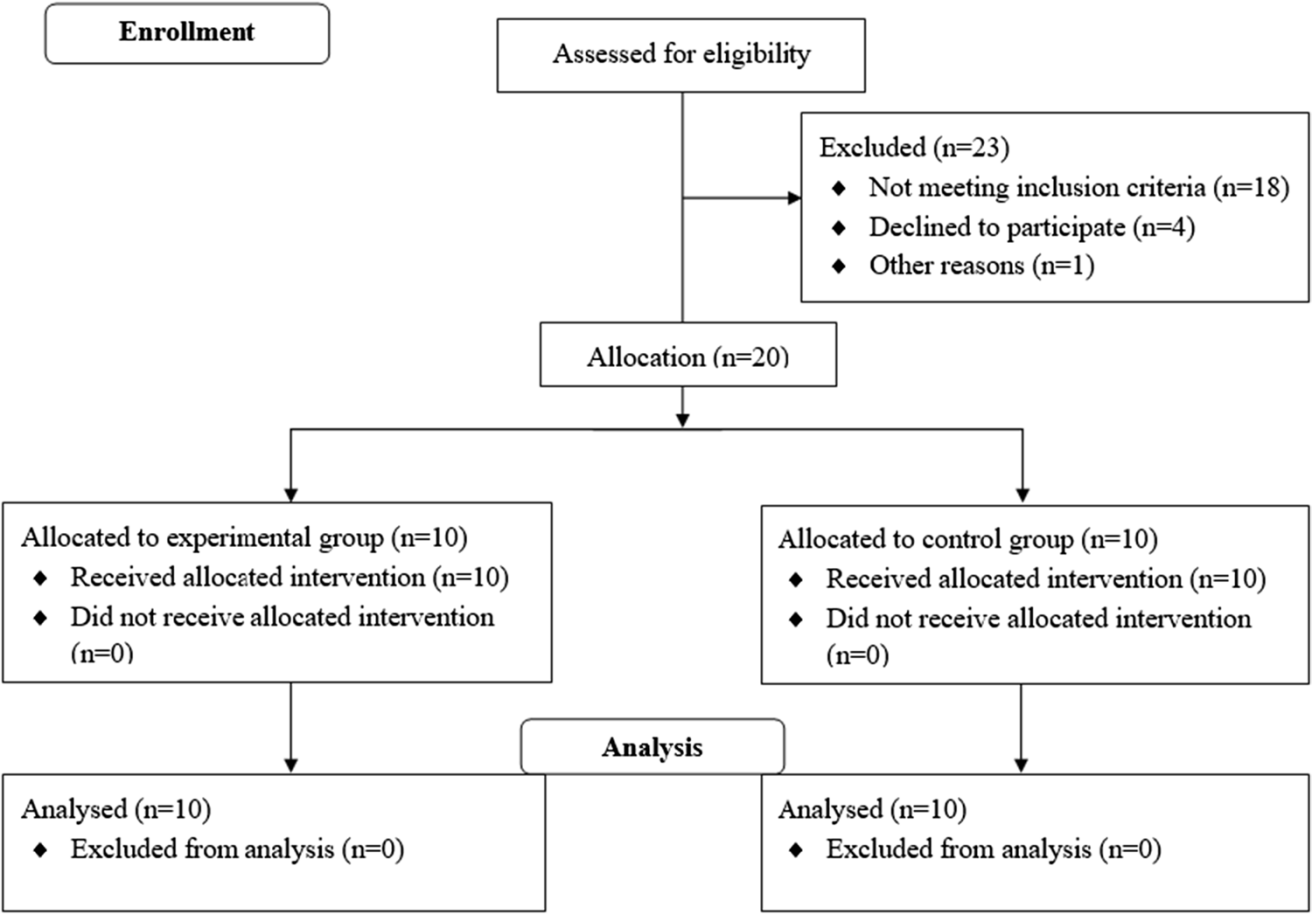
Study flowchart ( adapted from CONSORT 2010 Flow Diagram)

After excluding patients due to functional limitations to perform a physical activity program, or the presence of asthma, chronic obstructive pulmonary disease, hypothyroidism or cardiovascular diseases, 20 non-physically active women awaiting BS (BMI ≥ 45 kg·m^−2^; obesity class IV) voluntarily enrolled in the study (Fig. 1). Volunteers were recruited from two University Hospitals of an urban area serving ≈ 230,000 inhabitants.

Depending on their possibilities to attend the training program regularly, they were allocated to an experimental group (EG, *n* = 10) or a control group (CG, *n* = 10) (Table 1). Volunteers from both hospitals were distributed among both groups in order to have the most balanced group composition (EG: 6:4; CG: 5:5; hospital-1:hospital-2 ratio; 2 postmenopausal women per group).

**Table 1:** Baseline participant characteristics

	EG (*n* = 10)	CG (*n* = 10)	*p*
Age (years)	43 ± 5	42 ± 9	0.725
Weight (kg)	125.3 ± 13.9	115.8 ± 15.1	0.161
Postmenopausal (yes)	2 (20)	2 (20)	0.999
Medication (yes)	5 (50)	4 (40)	0.653
Diabetes (yes)	3 (30)	2 (20)	0.606
Smoker (yes)	1 (10)	1 (10)	0.166

Data are shown as number of cases (percentage) or mean ± *SD*. *p* values refer to differences between groups. *EG* experimental group, *CG* control group

### Intervention

Both groups followed the presurgical care indications from their corresponding medical team. Additionally, the EG performed a 12-week structured physical activity program (Table 2) at the University sports facilities under the supervision of Sport Sciences Graduates. Participants in the EG trained together, and the PAP alternated continuous aerobic exercise at individual Fatmax intensity with resistance training performed at low workloads. First, participants combined continuous aerobic training (CAT) at individual Fatmax intensity on a cycle ergometer and resistance training in a single session to familiarize themselves with regular physical exercise. During the 12 weeks of the training program, frequency progressed from 2 days/week (d/w) during the first month to 3 d/w during the second month, and then to 4 d/w until the end of the physical activity program (PAP). The sessions lasted 60 min for the first 2 months and 70 min for the third month. The single sessions of CAT at Fatmax started on the second month and progressed from 50 to 60 min. Intensity was monitored throughout the session by means of a pulsometer (POLAR H7 Bluetooth Smart, Polar Electro, Kempele, Finland). The actual time of the session that the subject spent at Fatmax was estimated from heart rate (HR) recordings. Resistance training (RT) was prescribed based on the percentage of one repetition maximum (1RM), which was determined using the Brzycki formula [22]. The exercises (quadriceps, hamstrings, latissimus dorsi, pectoral, deltoids, gastrocnemius, triceps, and biceps brachii) were performed using guided machines with a weight that ranged between 20% 1RM and 30% 1RM. The single RT sessions started on the second month with 1 d/w and progressed to 2 d/w the third month. Every session finished with five stretching exercises in standing position (quadriceps, hamstrings, pectoral, latissimus dorsi, and gastrocnemius).

**Table 2:** Physical activity program schedule

Type of exercise		Month		
		1	2	3
CAT at Fatmax	Weekly frequency (sessions/week)	2	2	2
	Volume (min)	40	50	60
	Intensity (W)	Fatmax	Fatmax	Fatmax
Resistance training	Weekly frequency (sessions/week)	2	1	2
	Volume (series × exer. × rep.)	2 × 5 × 20 20	3 × 8 × 30 50	4 × 8 × 30 60
	Volume (min)			
	Intensity (%1RM)	20	25	30
Stretching training	Weekly frequency (sessions/week)	2	3	4
	Volume (series × exer.)	1 × 5	1 × 5	1 × 5
	Duration (min)	1	1	1

*CAT* continuous aerobic training, *Fatmax* intensity at which maximal fat oxidation is registered, *min* minutes, *W* watts, *exer.* × *rep.* number of exercises performed and number of repetitions per exercise, *%1RM* percentage over a maximum repetition

### Measurements

Both groups were tested 1 week before the EG started the PAP and when the PAP ended (respectively PRE and POST). At both time points, anthropometry, resting metabolic rate, substrate oxidation during exercise, and physical fitness were measured. All the laboratory measurements were taken at ~ 80 m above sea, under controlled conditions (temperature 22–24 °C and relative air humidity 45–60%).

#### Anthropometry and Body Composition

Anthropometric measurements were taken between 7:00 and 9:00 A.M., after 12 h of overnight fasting, with an empty bladder [23], and a restriction of caffeine or alcohol intake and physical exercise during the previous 48 h. The ISAK protocol was used to measure both body weight (BW) and waist and hip circumferences [24], and BMI was calculated and expressed as kilograms per square·meter. Excess body weight both in kilogram (EBW) and in percentage (EBW%) were also calculated and expressed as raw or normalized values [25]. Visceral fat percentage (VF%), and fat mass and fat-free mass—both in kilogram and in percentage (FM and FFM vs FM% and FFM%, respectively)—were estimated by bioimpedance analysis [9] (Tanita BC-420MA, Tanita, Tokyo, Japan; tetra-polar, 50 kHz, 90 μA, 150-1200Ω, accuracy =  ± 2%).

#### Resting Metabolic Rate and Substrate Oxidation

An indirect calorimetry was performed immediately after the anthropometric measurements to estimate the resting metabolic rate (RMR) and the resting energetic substrate oxidation (fat oxidation (RFO) and carbohydrate oxidation (RCHO)) [26, 27]. Raw RMR and RFO values were also normalized both to body weight (RMR_BW_; RFO_BW_) and fat-free mass (RMR_FFM_; RFO_FFM_) for each participant.

Briefly, the participants rested in supine position for 30 min, while their respiratory gases were continuously analyzed (Oxycon Pro, Jaeger, Friedberg, Germany). Data of the last 10 min were used to calculate the RMR according to Weir’s equation [26], and substrate oxidation was calculated from non-protein respiratory quotient [27], using the following equations:

$$\begin{array}{c}\mathrm{RMR}=[\left(3.94\times {\mathrm{VO}}_{2}\right)+\left(1.106\times {\mathrm{VCO}}_{2}\right)]\times 1440\\ \mathrm{RFO}=(1.695\times {\mathrm{VO}}_{2})-(1.701\times {\mathrm{VO}}_{2})\\ \mathrm{RCHO}: (4.585\times {\mathrm{VO}}_{2})-(3.226\times {\mathrm{VO}}_{2})\end{array}$$

#### Cardiorespiratory Fitness Measurements and Determination of Fatmax

The participants performed two ergoespirometric incremental tests up to exhaustion on a cycle ergometer (Technogym Bike Med, Technogym, Gambettola, Italy) under continuous respiratory gas analysis (Oxycon Pro, Jaeger, Friedberg, Germany), separated by 48 h.

The first test was carried out immediately after the indirect calorimetry described above and it aimed to determine the peak oxygen uptake (VO_2_peak), the peak power output (PO_peak_), the peak heart rate (HR_peak_), and both ventilatory thresholds (VT1 and VT2). Participants performed a 5-min warm-up at 40 W with a controlled cadence at 60 revolutions per minute (rpm). Then, participants kept a voluntary cadence between 60 and 80 rpm while the power output (PO) increased 20 W every minute until volitional fatigue [24]. When inability to maintain a minimum of 60 rpm as a cadence occurred despite providing verbal encouragement, a 5-min active cool-down period started, in which participants pedaled at the cadence of the warm-up instructions. After that, a verification test was performed at a constant power of 100% of the maximal power registered during the incremental test. Participants were asked to maintain their previously voluntary cadence and pedal for as long as possible during the verification test until volitional fatigue, while verbal encouragement was provided. Lastly, a 5-min cool-down period was performed at 25 W. Verification test ≥ 60 s were used for analysis [28]. VO_2_peak was calculated as the average of the highest 30 s of VO_2_ and expressed in absolute values (VO_2_peak abs).

The MFO and Fatmax were determined in a second ergoespirometric test. This test was carried out in a fasting state. Participants started a 10-min warm-up at 20% of their PO_peak_ reached during the previous test, and then, in a first phase the PO increased by 10% PO_peak_ every 5 min until reaching 70% PO_peak_, or until respiratory exchange ratio (RER) reached 1.0. Then, a second phase started, and PO increased by 15 W every minute until exhaustion [24]. Participants were asked to maintain a cadence of 60 rpm during the warm-up and the first phase, while in the second phase they could modify it voluntarily from 60 to 80 rpm. Heart rate (HR) at Fatmax was registered and used to control the intensity of the aerobic exercise during the intervention (see below). An intermediate test was carried out in the EG on the sixth week of the PAP, solely to adapt the training loads depending on individual Fatmax changes.

Mean values of VO_2_ and VCO_2_ for the last 60 s in each 5-min step were needed to prescribe CAT at Fatmax intensity for each participant. First, fat oxidation (FO) rates were calculated according to Frayn’s equations [27], with the assumption that the urinary nitrogen excretion rate was negligible. Then, the identification of the maximal fat oxidation value measured in the second ergoespirometric test and its corresponding intensity in W were estimated using a third polynomial equation (P3 model) [29]. For these calculations, mean values for the last 60 s in each 5 min step were used.

HR values related to FO rates were also calculated for each participant to control the intensity during the intervention. The P3 model was used to construct another standard fitting curve for HR and FO values. HR at which Fatmax occurred was registered for each participant and used during sessions to ensure training at individual Fatmax intensity.

### Statistical Analysis

Continuous and categorical variables are presented as mean ± standard deviation (SD) and number of cases (percentage), respectively. The normal distribution of the continuous data was verified by the Shapiro–Wilk test, box plot, and Q-Q graphs. Student’s independent *t*-test, Mann–Whitney *U* test, and Fisher’s exact test were used for between-group comparisons at pre-intervention in normally distributed continuous variables, non-normally distributed continuous variables, and categorical variables, respectively. A two-way analysis of variance (ANOVA) of repeated measures with group (i.e., experimental vs. control) as between-subjects factor and time (i.e., pre- vs. post-intervention) as within-subjects factor was used to test the interaction between both factors. Skewed data were transformed before performing the ANOVA. Nonetheless, pre- and post-intervention values are shown in the original scale to facilitate their interpretation. All analyses were considered statistically significant at critical level of *p* ≤ 0.05. Moreover, partial eta squared (*η*^2^) was used as effect size index. The *η*^2^ magnitude was classified as trivial (< 10%), small (10–24%), medium (25–37%), and large (> 37%), respectively. In case of interaction between both factors (i.e., *p* ≤ 0.05 and/or *η*^2^ ≥ 10%), simple effects with Sidak correction for multiple comparisons were estimated for studying within-group changes. Standardized mean difference (*d*) was used as effect size index for pairwise comparisons. The *d* magnitude was classified as trivial (< 0.50), small (0.50–1.24), moderate (1.25–1.9), and large (≥ 2.00). All analyses were performed with the SPSS package program (version 25, SPSS Inc., Chicago, IL, USA).

## Results

### Anthropometry and Body Composition

Pre- and post-intervention values, as well as changes at follow-up based on the allocated groups and training effect in anthropometry and body composition, are shown in Table 3. Our findings showed that FM% at baseline was higher in the EG compared to the CG (*p* < 0.05, Student’s *t* test), while FFM% at baseline was lower in the EG compared to the CG (*p* < 0.05, Mann–Whitney test). No differences at baseline were noticed for the remaining analyzed variables. After the intervention, the results showed that FM% was reduced and FFM% increased only in the EG, and no changes at follow-up were found in the CG for any of the analyzed variables. However, compared with the CG, none of the analyzed variables at the 12-week follow-up changed in the EG.

**Table 3:** Effect of the intervention on anthropometric measurements

	Experimental (*n* = 10)		Change		Control (*n* = 10)		Change		ANOVA (interaction)		
Variables	Pre	Post	*p*	*d*	Pre	Post	*p*	*d*	*F*	*p*	*η*^2^ (%)
BMI (kg·m^−2^)^&^	48.7 ± 5.5	47.8 ± 5.9	0.071	− 0.86	44.2 ± 5.7	43.9 ± 5.2	0.540	− 0.28	0.837	0.372	4.4
Weight (kg)	125.3 ± 13.9	123.1 ± 14.8	0.078	− 0.84	115.8 ± 15.1	114.9 ± 13.9	0.497	− 0.31	0.690	0.417	3.7
EBW (kg)	60.9 ± 13.6	58.6 ± 14.4	0.078	− 0.84	50.2 ± 14.4	49.4 ± 13.2	0.497	− 0.31	0.690	0.417	3.7
EBW (%)	48.1 ± 5.8	47.1 ± 6.2	0.060	− 0.90	42.7 ± 6.8	42.4 ± 6.4	0.565	− 0.26	1.010	0.328	5.3
Fat mass (kg)^&^	66.3 ± 8.3	63.8 ± 8.5	0.006*	− 1.37	58.9 ± 8.8	58.6 ± 8.6	0.718	− 0.16	3.729	0.069	17.2
Fat mass (%)	52.9 ± 1.8	51.8 ± 2.2	0.015*	− 1.20	50.8 ± 2.4	50.8 ± 2.5	0.839	0.09	4.160	0.056	18.8
FFM (kg)	58.9 ± 6.3	59.2 ± 7.2	0.782	0.13	56.9 ± 7.5	56.3 ± 6.3	0.491	− 0.31	0.484	0.496	2.6
FFM (%)	47.1 ± 1.8&	48.1 ± 2.2	0.015*	1.20	49.2 ± 2.4	49.1 ± 2.5	0.839	− 0.09	4.160	0.056	18.8
Waist (cm)^&^	142 ± 14	140 ± 15	0.334	− 0.44	134 ± 16	134 ± 15	0.719	− 0.16	0.197	0.663	1.1
Hip (cm)	147 ± 11	146 ± 14	0.410	− 0.38	140 ± 14	139 ± 13	0.633	− 0.22	0.064	0.803	0.4
WHR (cm)^a^	0.9 ± 0.1	0.9 ± 0.1	0.721	− 0.16	0.9 ± 0.1	0.9 ± 0.1	0.929	− 0.04	0.037	0.850	0.2
Visceral fat (%)^&^	17.8 ± 2.5	17.2 ± 2.5	0.013*	− 1.23	15.6 ± 2.9	15.3 ± 2.7	0.189	− 0.61	0.944	0.344	0.5

*BMI* body mass index, *EBW* excess body weight, *FFM* fat-free mass, *WHR* waist-to-hip ratio. Data at pre- and post-intervention are delivered as mean ± SD

^&^Data were log-transformed before performing the ANOVA

^a^Non-normally distributed data

### Resting Metabolic Rate and Substrate Oxidation

**Fig. 2:**
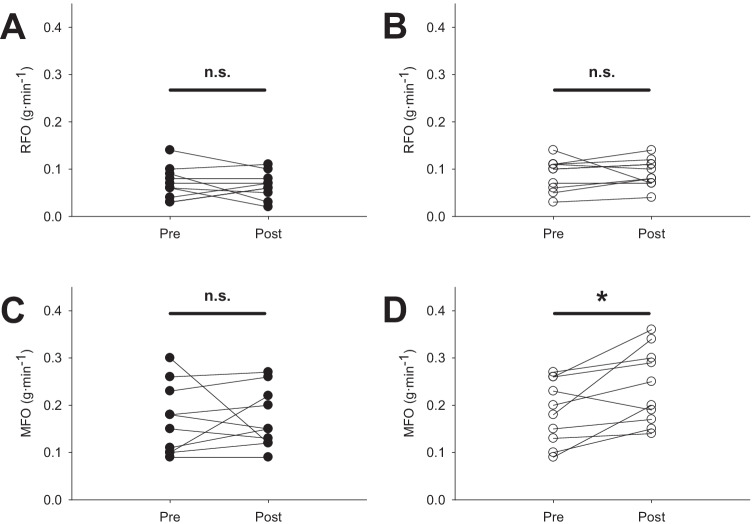
Individual data values of resting fat oxidation (RFO; **A** and **B**), and maximal fat oxidation at exercise (MFO; **C** and **D**). Left panels (filled circles) correspond to the control group. Right panels (open circles) correspond to the experimental group. Pre- and post- indicate that the measurement was taken before or after the intervention, respectively. The clear trend of increasing MFO specifically in the experimental group (**D**) can be seen. N.S: non-significant differences; *p < 0.05. For the mean and SD values, please see Tables 4 and 5

Pre- and post-intervention values, within-group changes at follow-up, and training effect in RMR and substrate oxidation are shown in Table 4. The results showed that RMR_FFM_ was higher in the EG compared to the CG (*p* < 0.05, Student’s *t* test), while no differences were found for the remaining variables at baseline. After the intervention, it was observed that RCHO increased in the CG, while no changes at follow-up or training effect were found in the EG for any of the analyzed variables. More specifically, RFO was unaffected by the intervention (Table 4, Fig. 2A, B).

**Table 4:** Effect of the intervention on resting metabolic rate and substrate oxidation

	Experimental (*n* = 10)		Change		Control (*n* = 10)		Change		ANOVA (interaction)		
Variables	Pre	Post	*p*	*d*	Pre	Post	*p*	*d*	*F*	*p*	*η*^2^ (%)
RCHO (g·min^−1^)^&^	0.121 ± 0.06	0.122 ± 0.05	0.851	0.09	0.102 ± 0.08	0.143 ± 0.08	0.008*	1.35	3.969	0.062	18.1
RCHO (g·day^−1^)^&^	173.3 ± 81.4	175.0 ± 67.7	0.826	0.10	146.1 ± 119.1	206.7 ± 110.5	0.006*	1.38	4.098	0.058	18.5
RCHO (%)	53.2 ± 20.9	53.7 ± 14.4	0.932	0.04	51.6 ± 26.3	62.7 ± 18.5	0.067	0.87	1.738	0.204	8.8
RFO (g·min^−1^)	0.088 ± 0.03	0.092 ± 0.03	0.685	0.18	0.070 ± 0.033	0.065 ± 0.03	0.612	− 0.23	0.432	0.520	2.3
RFO (%)	46.7 ± 20.9	46.3 ± 14.4	0.932	− 0.04	48.3 ± 26.3	37.2 ± 18.5	0.067	− 0.87	1.738	0.204	8.8
RFO_BW_	0.710 ± 0.26	0.731 ± 0.19	0.804	0.11	0.622 ± 0.28	0.630 ± 0.20	0.924	0.04	0.012	0.913	0.1
RFO_FFM_	1.5 ± 0.5	1.5 ± 0.3	0.964	0.02	1.2 ± 0.5	1.2 ± 0.3	0.852	0.08	0.010	0.921	0.1
RFO (g·day^−1^)	128.6 ± 49.6	130.5 ± 40.1	0.895	0.06	98.3 ± 49.1	97.0 ± 41.1	0.928	− 0.04	0.025	0.875	0.1
RMR (kcal)	1869 ± 406	1894 ± 336	0.827	0.10	1480 ± 474	1695 ± 270	0.073	0.85	1.414	0.250	7.3
RMR_BW_ (kcal)	14.8 ± 2.5	15.3 ± 2.0	0.582	0.25	12.42 ± 3.2	14.4 ± 1.8	0.040*	0.99	1.359	0.259	7.0
RMR_FFM_ (kcal)	31.7 ± 6.1	32.0 ± 4.6	0.859	0.08	24.6 ± 5.4	29.2 ± 3.7	0.029*	1.06	2.407	0.138	11.8

*RCHO* resting carbohydrate oxidation, *RFO* resting fat oxidation, *RMR* resting metabolic rate, *BW* body weight, *FFM* fat-free mass. Data at pre- and post-intervention are delivered as mean ± SD

^&^Data were log-transformed before performing the ANOVA

^a^Non-normally distributed data

### Cardiorespiratory Fitness Measurements and Substrate Oxidation During Exercise

Pre- and post-intervention values, changes at follow-up based on the allocated groups, and training effect in cardiorespiratory fitness and substrate oxidation are reported in Table 5. The findings showed that VO_2_peak abs/BW at baseline was higher in the CG compared to the EG (*p* < 0.05, Student’s *t* test), and no differences were found between the EG and the CG at baseline for the remaining cardiorespiratory and substrate oxidation variables.

**Table 5:** Effect of the intervention on cardiorespiratory fitness and substrate oxidation during exercise

	Experimental (*n* = 10)		Change		Control (*n* = 10)		Change		ANOVA (interaction)		
Variables	Pre	Post	*p*	*d*	Pre	Post	*p*	*d*	*F*	*p*	*η*^2^ (%)
VO_2peak abs_ (L·min^−1^)	1.8 ± 0.4	1.9 ± 0.4	0.073	0.85	1.7 ± 0.2	1.8 ± 0.2	0.265	0.51	0.281	0.602	1.5
VO_2peak abs/BW_ (mL*BW^−1^·min^−1^)	14.6 ± 3.2	15.7 ± 2.8	0.166	0.65	21.6 ± 8.4	22.6 ± 8.7	0.204	0.59	0.008	0.931	0.0
VO_2peak abs/FFM_ (mL*FFM^−1^·min^−1^)	31.1 ± 6.5	32.7 ± 5.4	0.119	0.73	25.2 ± 9.4	26.3 ± 9.7	0.262	0.52	0.114	0.739	0.6
PO_peak_ (W)	127 ± 41	148 ± 46	< 0.001*	2.36	146 ± 23	148 ± 25	0.621	0.22	11.400	0.003*	38.8
VT2 (W)^a^	104.4 ± 24.1	128.6 ± 39.6	0.001*	1.82	98.5 ± 17.1	114.0 ± 18.9	0.018*	1.17	1.013	0.328	5.6
VO_2peak abs/BW_ at VT2 (L·min^−1^)	12.5 ± 2.7	13.8 ± 3.7	0.064	0.93	11.8 ± 1.9	13.6 ± 1.1	0.011*	1.27	0.270	0.610	1.6
MFO (g·min^−1^)	0.187 ± 0.068	0.239 ± 0.080	0.025*	1.10	0.170 ± 0.074	0.171 ± 0.063	0.963	0.02	2.888	0.106	13.8
MFO_BW_ (mg·kg^−1^·min^−1^)	1.4 ± 0.4	1.9 ± 0.6	0.020*	1.14	1.4 ± 0.6	1.4 ± 0.4	0.922	− 0.04	3.522	0.077	16.4
MFO_FFM_ (mg·kg^−1^·min^−1^)	3.1 ± 0.9	4.0 ± 1.2	0.044*	1.21	2.9 ± 1.1	2.9 ± 0.9	0.790	0.14	2.867	0.108	13.7
Fatmax (W)^a^	37.1 ± 10.2	49.2 ± 12.7	< 0.001*	2.15	42.2 ± 9.3	38.7 ± 9.5	0.181	− 0.62	19.223	< 0.001*	51.6
PO at Fatmax (%)	23.5 ± 5.3	31.6 ± 7.0	< 0.001*	2.40	26.1 ± 5.6	24.6 ± 5.5	0.320	− 0.46	20.440	< 0.001*	53.2
VO_2peak_ at Fatmax (%)^a^	54.9 ± 8.8	55.9 ± 7.2	0.679	0.19	50.9 ± 8.0	50.9 ± 9.1	0.955	− 0.03	0.114	0.739	0.6
HR at Fatmax (%)	77.1 ± 7.9	79.6 ± 6.8	0.347	0.43	71.9 ± 12.7	71.8 ± 7.5	0.967	− 0.02	0.508	0.485	2.7

*VO*_*2*_ oxygen uptake, *PO* power output, *MFO* maximal fat oxidation, *Fatmax* intensity at which maximal fat oxidation was reached, *HR* heart rate, *BW* body weight, *FFM* fat-free mass, *VT2* second ventilatory threshold. Data at pre- and post-intervention are delivered as mean ± SD

^&^Data were log-transformed before performing the ANOVA

^a^Non-normally distributed data

Regarding cardiorespiratory fitness, it was observed that Fatmax, VT2, PO at Fatmax, and PO_peak_ significantly increased only in the EG. Similarly, MFO during exercise only increased in the EG (Table 5, Fig. 2C, D).

## Conclusion

This study aimed to describe the effects of a training program that combined aerobic exercise at Fatmax and low-intensity resistance training in middle-aged women affected by class IV obesity while they were awaiting BS. To the best of our knowledge, this is the first study in which the effects of an individualized and supervised PAP of these characteristics has been carried out on this population. As expected, the results showed that in the EG, the body composition and the physical fitness improved. Interestingly, in addition to that, an increase in fat oxidation specifically during exercise, but not at rest, was also found in this group and not in the control group.

Previous studies in which exclusively aerobic exercise at Fatmax was prescribed to overweight and obese women have shown reductions of both FM and FFM [16–18], although resistance training was not included in these studies. The results of the current study suggest that including resistance training prevents FFM reduction. This finding could be of clinical interest, since achieving a stable or improved FFM has been associated to better weight loss and prevention of weight regain [6, 8, 9] in the specific context of BS. This can be related to the increased RMR associated to the FFM maintenance that has been previously described in BS patients [30, 31]. The observations in the specific population that participated in the present study reinforce this idea, and suggest that FFM maintenance in patients waiting for BS is important to counteract the deleterious effects on resting VO_2_ and energy expenditure that have been associated with the severe diet-induced weight loss commonly prescribed to these patients [12].

As mentioned earlier, one of the most remarkable findings was the increase of fat oxidation during exercise. This observation agrees with previously published data in middle-aged overweight women [17], therefore suggesting that fat oxidation during exercise may be sensitive to exercise-based interventions, independently of the obesity level. The increase of either MFO and Fatmax, in response to exercise training, is currently considered an indicator of metabolic flexibility [32]. After the intervention, MFO increased in the EG. Work capacity also increased. Taken together, these results may reflect a higher work efficiency after the PAP. Previous works have suggested that this finding could be only observed associated to body weight reductions and low levels of muscle work [12, 33]. However, given the fact that in our results these responses were not associated to weight changes, this finding may be interpreted as that the PAP described here was able to restore the impaired metabolic flexibility that has been suggested is associated to obesity [13]. The confirmation of this hypothesis, as well as the identification of its explanatory mechanisms at the molecular or cellular level, needs further experimentation. In this regard, acute-phase reactants like serum amyloid A [34] and adipokines like leptin [35, 36] are potential targets that merit future investigation.

Some limitations should be considered in this research. First, the small size of the sample and the short period of the intervention make the generalization of these findings difficult, especially due to the absence of a retention test. These circumstances are highly common in patients awaiting BS since they follow several medical controls which directly affect their possibilities to participate regularly in long interventional trials carried out in ecological environments. Second, cycling was considered the best choice to prevent knee pain instead of a treadmill due to the BMI of the participants, but it caused discomfort in some of the participants when performing CAT for more than 30 min. Although the intervention allowed 1-min breaks in the middle of the Fatmax sessions, it was not enough to avoid this problem in all cases as severely obese adults often suffer from exercise intolerance [37]. Third, training sessions were carefully programmed and controlled but the measurements were not sufficient to evaluate molecular changes in muscle tissue related to substrate oxidation. This fact is highly relevant as skeletal muscle contributes to VO_2_, energy expenditure, and FO, so knowing oxidative enzyme content could be useful to design future interventions to stimulate non-aggressive and progressive weight loss in patients awaiting BS. In addition, MFO and Fatmax depend on several key factors such as training status, sex, or chronic nutritional status [14], so these findings should be analyzed carefully since no similar studies are available nowadays in middle-aged women awaiting BS. Therefore, future studies could propose similar interventions for this population in which other training modalities may be performed and accurately controlled to solve these limitations. Last, but not the least, although the bioimpedance system that we have used in this and in previous works with this population [9, 38] has been validated and is sensitive to body composition changes [39], more precise methods to measure body composition would be preferred in future studies. In addition, the potential effect of some covariables—such as age, menopausal state, and medications—should be also analyzed.

Despite these limitations, our work adds some novelties and strengths to previous published research. First, the protocol used to determine the Fatmax and MFO has been applied only in men with lower BMI than those recorded in our sample [24]. Second, we included a verification step at the end of the protocol to ensure its maximality [28]. This is especially relevant in a population with very low tolerance to exercise, such the one described here. Third, ergoespirometry were performed to every participant, therefore allowing for individual measurement of Fatmax, W, and HR based on which the exercise intensity was individually prescribed. Other previous works, for example, have extrapolated the target HR to reach during the exercise session by the whole sample, simply by measuring a subset of participants and estimating the mean value [40]. In addition to that, in our experimental design, the ergoespirometries were performed in a clycoergometer, which provided a greater transfer of the obtained values to the PAP. Therefore, our experimental design was able to overcome some limitations of previous studies. Finally, we consider that similarly to other previous works [9, 38], the individualized approach that we followed with every participant was a key factor to reach the high adherence to the PAP during the 3 months of the intervention. This is particularly noticeable for a population like this one, with very low intrinsic motivation to exercise as well as multiple socioeconomic and sociocultural limiting factors to be physically active.

Albeit this is a first description of the effects of combining aerobic exercise at Fatmax with low-intensity resistance training, these results may be of clinical relevance, since patients awaiting BS are typically requested to lose from 5 to 10% body weight before the intervention to decrease the associated surgical risk [41]. To reach this reduction, highly restrictive dietary protocols are usually prescribed aimed to change the patient’s metabolism [41]. So, if an individualized and supervised PAP such the one described here might be able to stimulate higher fat oxidation rates during exercise, while maintaining FFM and RMR in the long term, its implementation before surgery may counteract some of the unwanted side effects of the highly restrictive dietary interventions usually seen in this population [42].
